# Cervical approach to cervico-mediastinal goiters: Experience of a Moroccan ENT tertiary center - Case series

**DOI:** 10.1016/j.amsu.2021.01.081

**Published:** 2021-01-26

**Authors:** Y. Oukessou, M.A. Mennouni, L. Douimi, S. Rouadi, R.L. Abada, M. Roubal, M. Mahtar

**Affiliations:** ENT Head and Neck Surgery Department, Ibn Rochd University Hospital, Faculty of Medicine and Pharmacy, Hassan II University, Casablanca, Morocco

**Keywords:** Cervicomediastinal, Substernal, Goiter, Cervical approach, Case series

## Abstract

**Background:**

The purpose of the study was to analyze and discuss the demographic, clinical, radiological, therapeutic and postoperative findings of the Cervico-mediastinal goiters (CMG) treated through a cervical approach admitted in the ENT department of Ibn Rochd university hospital, Casablanca, Morocco between January 2014 and January 2020.

**Materiels and methods:**

Over a period of 6 years, 116 patients underwent surgical treatment for CMG. It was defined as a goiter extending below the plane of superior thoracic aperture on CT scan. All our patients had clinical, biological and radiological assessment before surgery. A nasofibroscopy was carried out pre and postoperatively. All the CMG have been extracted trough a cervical approach by an experimented ENT surgeon.

**Results:**

84,48% of the CMG was diving into the anterior mediastinum and 15.52% into the posterior. The CMGs extended above, at, and below the level of the aortic arch respectively in 76.72%, 18.10% and 5.17% of the patients. All of 116 goiters were successfully removed through a cervical approach. No patient required a sternotomy. Postoperatively, vocal cord paralysis was transient in 3 patients (2.58%) and permanent in 2 patients (1.72%). Hypocalcemia was transient in 10 patients (8.62%) and permanent in 2 patients (1.72%). Final histology found 106 benign multinodular goiters (91.37%), 7 papillary carcinomas (6.03%) and 3 vesicular carcinomas (2.58%). No death was noted.

**Conclusions:**

With expertise in thyroid surgery, cervical approach for CMGs is safe and sufficient in the majority of the cases with low morbidity rate and no mortality.

## Highlights

• In the literature, there is no consensus on the definition of cervico-mediastinal goiters.
• CMG is a challenging disease that needs proper clinical, biological and radiological assessment.
• Malignancy in a CMG is not higher than that of the cervical goiters and most of the foci in CMG were in the intrathoracic region.
• With expertise in thyroid surgery, cervical approach for CMGs is safe and sufficient in the majority of the cases.

## 1 Introduction

Cervico-mediastinal goiter (CMG) can be defined as an enlarged cervical thyroid gland with a component extending into the mediastinum. Secondary mediastinal, substernal, retrosternal, cervicothoracic, diving, plunging goiters are other terms used in published literature. In contrast, primary mediastinal goiter, is defined as an ectopic thyroid tissue with no continuity with the cervical thyroid [1], which is a very rare entity that accounts for only 0.2–1% of all the intrathoracic goiters [2] (see Table 1, Table 2, Fig. 2, Fig. 3)

**Table 1:** Patient characteristics with cervicomediastinal goiter and postoperative complications.

Characteristics	No of patients (n = 116)
**Age**	47,6 (18–77)
**Sex**	
Female	92 (79.31%)
Male	24 (20.69%)
Ratio	3.8
**Symptoms at presentation**	
Cervical mass	116 (100%)
Dyspnea	47 (40.5%)
Dysphagia	14 (12.07%)
Dysphonia	7 (6.03%)
Paroxysmal coughing	3 (2.58%)
Superior vena cava syndrome	2 (1.72%)
Hyperthyroidism	10 (8.62%)
Hypothyroidism (0.86%)	1 (0,86%)
Cervical lymphadenopathies	6 (5.17%)
**Surgical procedure**	
Cervical approach	116 (100%)
Extra-cervical approach	0
Total thyroidectomy	105 (90.52%)
Hemithyroidectomy	11 (9.48%)
Neck dissection	6 (5.17%)
**Histology**	
Benign multinodular goiters	106 (91.37%)
Papillary carcinomas	7 (6.03%)
Vesicular carcinomas	3 (2.58%)
**Vocal cord movement impairment**	
Unilateral permanent paralysis	2 (1.72%)
Unilateral transient paralysis	3 (2.58%)
Bilateral paralysis	0
**Hypocalcemia**	
Transient	10 (8.62%)
Permanent	2 (1.72%)
**Wound issues**	
Compressive hematoma	1 (0.86%)
Infection	0
Tracheomalacia	0
**Median length of stay**	3.0 (2–16)

**Table 2:** Preoperative CT findings (n = 116).

Anterior mediastinal extension	84,48% (n = 98)
Posterior mediastinal extension	15.52% (n = 18)
Unilateral mediastinal extension	80.17% (n = 93)
Right side	49.13% (n = 57)
Left side	31.04% (n = 36)
Bilateral mediastinal extension	19.83% (n = 23)
Extension Above the level of aortic arch	76.72% (n = 89)
Extension to the level of aortic arch	18.10% (n = 21)
Extension below the level of aortic arch	5.17% (n = 6)

**Fig. 1:**
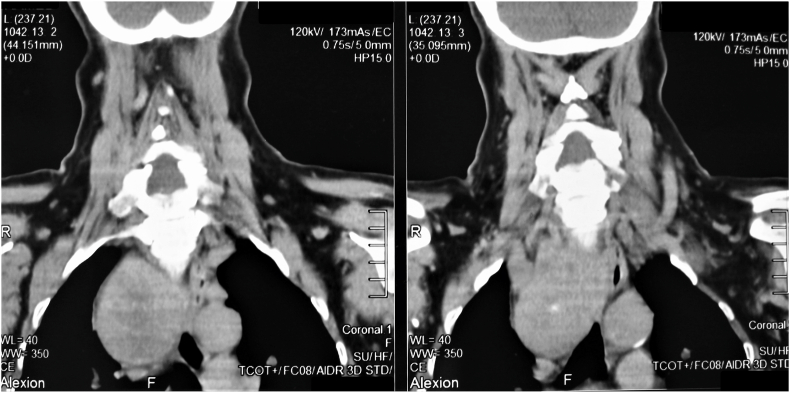
CT scan of a CMG extending below the level of the aortic arch and reaching the level of carina on the right side.

**Fig. 2:**
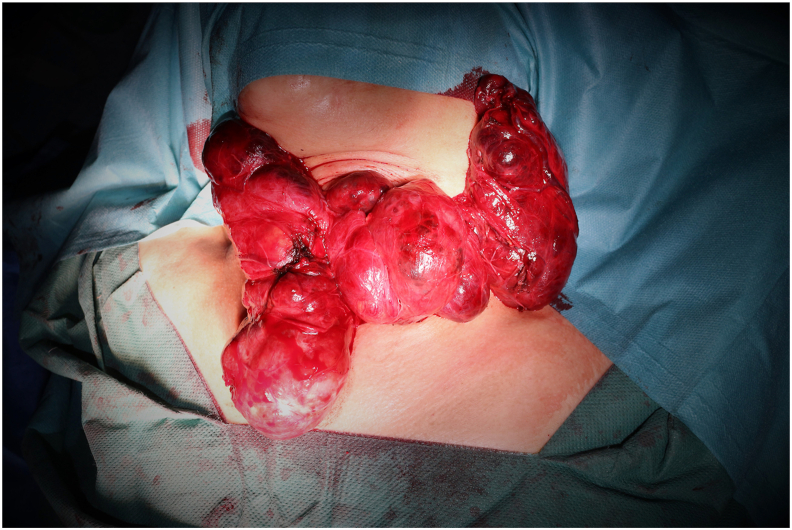
Per-operative image showing the extraction of the CMG shown in Fig. 1 through a cervical approach.

**Fig. 3:**
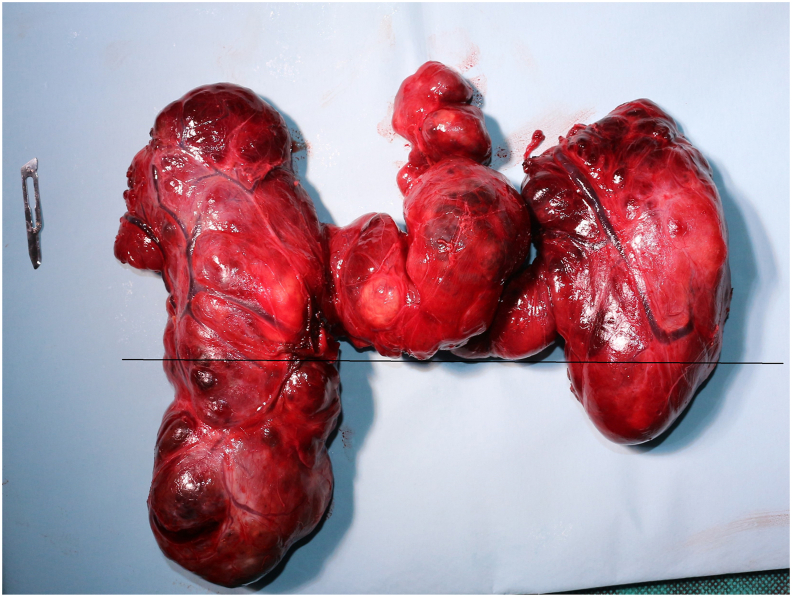
Enbloc excision of the CMG (the line shows the limit between the cervical and thoracic components).

A CMG can be found unilaterally or bilaterally and is mostly located in the anterior mediastinum, only 10%–15% of substernal goiters are located in the posterior mediastinum [3].

In the present study, we report, through a series of 116 cases CMGs, the experience of the ENT department of a Moroccan tertiary center in the management of this entity through cervical approach. Epidemiological, clinical, paraclinical, histological and postoperative findings were retrospectively analyzed.

This work has been reported in line with the PROCESS Guidelines 2020 [4].

## 2 Methods

Between January 2014 and December 2019, 1450 patients had thyroid surgery at the ENT/head and Neck surgery department of the Ibn Rochd university hospital, Casablanca, Morocco. 116 (8%) of them underwent a thyroidectomy for cervico-mediastinal goiter (CMG). A CMG was defined as a goiter extending below the plane of superior thoracic aperture on CT scan [5] that was realized systematically when the inferior border of one of the lobes couldn't be palpated clinically.

Demographic, clinical, radiological, surgical, and follow-up data were collected from clinical case notes. Patients with ectopic thoracic goiter were excluded.

pre- and postoperatively, a laryngopharyngeal flexible endoscopy was performed in order to evaluate the vocal cords mobility. Standard biological parameters (TSH, fT3, fT4 and serum calcium levels), chest X-ray, cervical ultrasonography (US) and a cervicothoracic CT scan were performed preoperatively in all patients. No MRI was carried out.

All surgeries were done by a senior ENT Professor, with high expertise in thyroid surgery (more than 15 years of experience), under general anesthesia with tracheal intubation insured by an anesthesiologist experienced in awake fiber optic intubation. Incision consisted in all cases in a Kocher incision. Superior and inferior subplatysmal flaps were elevated. The median raphe was opened using monopolar cautery. The procedure was always initiated on the less voluminous and plunging side. The infrahyoid muscles were sectioned and retracted laterally only when the extraction of the lobe was laborious. Extracapsular dissection was performed with ligation of the feeding vessels in order to reduce thyroid vascularization and therefore decrease its volume and tension. The extraction of the thoracic component was done using Blunt finger dissection and progressively and gently pulled out of the mediastinum. The laryngeal nerves and the parathyroid glands were systematically identified and spared along with their vascular supply. Perfect hemostasis was ensured at the end of the intervention and the wound was closed after setting up a drain tube system in both lodges. In case of advanced age, comorbidities or important tracheal compression, a 24–48 h stay in the ICU was necessary. Post-operative calcium and parathormone levels were measured only if there were hypocalcemia clinical signs. Hypocalcemia was defined as a corrected calcium concentration inferior to 2.2 mmol/l in a symptomatic patient. Vocal cord paralysis and hypoparathyroidism were considered permanent if they persisted at 6 months after surgery. Postoperatively, the patients received 7 days long prescription of antibiotics and antalgics, calcium supplementation was not prescribed in a systematic fashion.

## 3 Results

Among the 116 patients operated for cervico-mediastinal goiter in our institution, 92 were women (79,31%) and 24 were men (20,69%), the female to male ratio was 3.8:1. The median age was 47,6 years (range 18–77 years). The most affected age group was between 38 and 47 years, it represented 38,79% of all patients. No patient has experienced a thyroid surgery before, one patient had amiodarone-induced hypothyroidism.

All patients showed a cervical mass, 73 patients (63%) presented compressive symptoms at the time of the surgery. Dyspnea was the leading symptom (40.5%), followed by dysphagia (12.07%), dysphonia (6.03%), Paroxysmal coughing (2.58%) and superior vena cava syndrome (1.72%). At presentation, 11 patients (9,48%) had clinical signs of dysthyroidism which were confirmed biologically, 10 patients with hyperthyroidism (8.62%) and one patient with hypothyroidism (0.86%). These patients have been operated after that euthyroidism was restored by the endocrinologists. 6 patients (5.17%) presented cervical lymphadenopathies.3 patients (2.59%) presented unilateral vocal cord paralysis and 4 patients unilateral vocal cord paresis (3.44%). The mean duration of symptoms was 10 years (range 2–25 years). The ultrasonography was able to detect the diving nature of the goiter in only 24 patients (20.69%) whereas the X-Ray showed a cervical opacity extended to the thorax in 85 patients (73.27%), a tracheal deviation in 72 cases (62.07%), which was particularly obvious in unilateral and asymmetric goiters, and a tracheal stenosis was seen in 5 cases (4.31%).

CT scan has demonstrated that in 23 cases (19.83%) both lobes were plunging and in 93 cases (80.17%) only one lobe was plunging. It was the right lobe in 57 cases (49.13%) and the left in 36 cases (31.04%). The CMG involved the anterior mediastinum in 98 cases (84.48%) and the posterior mediastinum in 18 cases (15.52%). The mass was located above, at the level and inferiorly to the level of the aortic arch in respectively 89 cases (76.72%), 21 cases (18.10%) and 6 cases (5.17%).

None of the patients of this series required a sternotomy, all of them underwent thyroid surgery through a cervical approach (n = 116, 100%). Total thyroidectomy was realized in 105 patients (90.52%) and hemithyroidectomy in 11 patients (9.48%). All the 6 patients who presented cervical lymphadenopathies underwent lateral and central therapeutic cervical neck dissection, all of them revealed a papillary thyroid carcinoma. The anatomopathological diagnoses included 106 benign multinodular goiters (91.37%), 7 papillary carcinomas (6.03%) and 3 vesicular carcinomas (2.58%). The mean duration of stay at the hospital was 3 days. A single patient presented a postoperative compressive hematoma that required an urgent drainage with hemostasis of the responsible vessels. 2 patients (1.72%) presented a permanent and 3 patients (2.58%) a transient unilateral vocal fold paralysis. The latters have shown a total recovery within 6 months of the operation. No patient presented bilateral paralysis. Hypocalcemia was transient in 10 patients (8.62%) and permanent in two patients (1.72%). No case of postoperative infection, tracheomalacia or death were noted.

## 4 Discussion

In the literature, there is various different definitions suggested of the CMG. The one we based our study on, is the extension of the thyroid gland below the thoracic inlet. Other definitions include goiters of which at least 50% of their volume is in the chest, goiters reaching the aortic arch, goiters with extension below the fourth thoracic vertebra or a portion of the thyroid gland that remains retrosternal at the clinical examination [6].

RSG incidence is reported to be between 1.7% and 45% of thyroidectomies [7]. This is probably due to the lack of standardization in the definition [8].

Like goiter itself, CMG occurs most commonly in the fifth decade of life. Women are affected much more frequently than men, with the reported ratio ranging from 5 to 9:1 [9]**.** In our series, the ratio was 3.8:1 and the mean age 47,6.

The most frequent reason for consultation was the cervical swelling followed by signs of compression. Signs of tracheal compression are first and foremost, dominated by dyspnea [10]. Most often, it is intermittent, on exertion or orthopnea [11]**.** It was found in 40.5% of our cases.

The tracheal compression can take an acute form secondary to a sudden increase in the volume of the gland by an intracystic hemorrhage or a degeneration which can lead to asphyxia. This vital respiratory emergency picture is present in 0.8% and 6.4% of cases respectively in the series of Torre and Fadel [12,13]. None of our patients presented with acute respiratory distress.

The esophageal compression was the second most frequent compression sign in our series (12.07%). It most often results in dysphagia. It is usually late, more or less marked, progressive, predominates in solids and can also be associated with pharyngolaryngeal reflux [14]**.**

Its incidence in the literature is between 5 and 20% [15]**.** The incidence of laryngeal nerve impairment varies in the literature between 4.7% [16] and 10% [14]**.**

It must be noted that while benign disease affecting the recurrent laryngeal nerve is rare, compressive forces in large volume disease may impact neural function or compromise perineural vasculature. The resultant dysphonia manifest from such an insidious process may not be overtly obvious to the patient nor the clinician. Therefore, particular care must be employed in the thorough examination of laryngeal function prior to undertaking surgery for CMG [6]**.**

Less frequently are observed compressive effects on cervical and mediastinal neurovascular structures with rare cases of superior vena cava syndrome due to venous compression and thrombosis [2]. Its rate ranges from 3% to 19%. In our series, 2 patients (1.72%) had this complication, both of them were treated successfully via cervical approach.

Chest X-rays can demonstrate a mediastinal opacity and a tracheal deviation and/or stenosis predicting a difficult intubation [17]. Ultrasound has a relatively small role in evaluating the substernal goiter as it is not very effective in the substernal position [18]**.**

A CT scan is the preoperative radiological test considered as the gold standard [5]**.** The extensions inferior to the level of the aortic arch or to the posterior mediastinum seem to be the situations that are at major risk of an extracervical approach [5], as well as the loss of normal fascial planes between portions of the goiter and the surrounding mediastinal structures suggesting the presence of adherences [19]**.**

There were 18 patients in this series with an extension into the posterior mediastinum, all of them have been successfully extracted through a cervical approach alone without morcellation.

Nonsurgical treatment of a substernal goiter with thyroid hormone or radioactive iodine ablation is almost always unsuccessful; in addition, attempted radioactive ablation can sometimes precipitate respiratory distress, especially in elderly patients [20]**.**

Because of the risk of compressive complications, malignancy and even sudden death; the surgical excision (total thyroidectomy with enbloc removal of the intrathoracic portion of the thyroid) is the treatment of choice even in the absence of clinical signs [2]**.**

The vast majority of CMG can be safely resected through a cervical incision although a combined cervicothoracic approach may be necessary in up to 2% of cases [19]**.**

Morcellation has to be avoided in order to lessen the possibility of bleeding and because of the possible presence of an occult carcinoma within the gland [21]**.**

The vast majority of CMG goiters originates from the lower part of one lobe or both lobes of cervical thyroid or isthmus and grow down through the thoracic inlet under the action of swallowing, gravity and thoracic negative pressure. Because of this cervical origin, blood supply is insured by the inferior thyroid artery and its branches, therefore the cervical extraction is possible and safe [2]. At the contrary, Ectopic thyroids draw their blood supply from the aorta, innominate or internal thoracic arteries and may lack a cervical component altogether [21]**.**

In our experience, the preoperative assessment of the mobility of the cervical component of the goiter at the clinical examination is of paramount importance. The extraction through a cervical approach is more likely to be possible when the mobility is conserved which was the case for all our patients.

A recent study by Sahbaz et al. showed that the incidence of malignancy in a CMG is not higher than that of the cervical goiters and most of the foci in CMG were in the intrathoracic region [22]. This finding underlines the limits of the US and FNA in the assessment of the malignancy risk in the CMGs. In this study 19% of the cases presented a papillary carcinoma. In a systematic review (23 case series), malignancy rate in retrosternal goiter was defined between 3.7 and 22.6% [23]**.** In our study, a malignant neoplasia was diagnosed in 8.62% of cases.

The utility of manubriotomy, or sternal split, has long been a dilemma in surgery for the SSG. While providing unparalleled exposure to the infra-cervical disease, it is a procedure imparting significant morbidity on the patient [6]**.**

Testini et al. in two large multicenter studies demonstrated that the risks of permanent hypoparathyroidism and both transient and permanent RLN palsy are higher when the CMG is approached extracervically compared to the traditional transcervical approach [24,25]**.**

In our experience, all the CMG have been resected through cervical approach. There was no mortality with a low morbidity which was no different than seen in thyroidectomies for cervical goiters. This approach, that seems to be safe, requires adequate experience at first in cervical and then in cervico-mediastinal goiters resection procedures. The training needs to be progressive, beginning with low volume and slightly plunging goiters into the anterior mediastinum, and slowly extend to bigger goiters, more plunging and extending into the posterior mediastinum. At every stage of the learning process and specially in the beginning, the involvement of the thoracic surgeon is mandatory.

Limitations of the present study include the inherent biases in retrospective studies.

## 5 Conclusion

In Morocco, the thyroid pathology represents a real public health problem, in some regions with high prevalence of iodine deficiency, the prevalence of goiters arises to 50% of the population. Subsequently, thyroid surgery is performed in a daily basis in our institution. In the case of secondary CMG, the review of our experience confirms that thyroidectomy can be carried out safely, by ENT surgeon with high expertise, through cervical approach, with very low morbidity, even in case of presence of respiratory, neurologic and vascular compressions or extension into the posterior mediastinum. These findings confirm the results of a previous study conducted in our department [26].

## Declaration of competing interest

None.

## Registration of research studies

Name of the registry: Research registry.

Unique identifying number or registration ID: 6401.
